# Mode of delivery among pregnant people with obesity undergoing labor induction in the late preterm period

**DOI:** 10.1002/pmf2.70122

**Published:** 2025-10-08

**Authors:** Daniella Rogerson, Minhazur Sarker, Alice Sutton, E. Nicole Teal, Cynthia Gyamfi‐Bannerman

**Affiliations:** ^1^ Department of Obstetrics Gynecology and Reproductive Sciences University of California San Diego La Jolla California USA

## Abstract

**Introduction:**

Pregnant people with obesity are more likely than those without obesity to undergo induction of labor and to require unplanned cesarean delivery during induction at term. Whether they are more likely to undergo unplanned cesarean delivery during preterm induction of labor is unknown. This study examines the induction of labor success among people with obesity undergoing indicated late preterm induction of labor.

**Methods:**

This is a secondary analysis of a multicenter, randomized trial of betamethasone versus placebo among pregnancies at risk for late preterm delivery, defined as delivery between 34 weeks and 0 days and 36 weeks and 6 days between 2010 and 2015. This study included pregnant people with live singleton nonanomalous gestations at high risk for late preterm delivery before 36 weeks and 6 days. This secondary analysis included all participants who underwent a medically indicated preterm induction of labor starting before 36 weeks and 5 days. We excluded participants who underwent planned cesarean delivery, had spontaneous labor, or had a body mass index (BMI) <18.5. We compared final delivery mode among participants undergoing preterm induction stratified by obesity class: no obesity (BMI 18.5–29.9), class 1 obesity (BMI 30–34.9), class 2 obesity (BMI 35–39.9), and class 3 obesity (BMI ≥ 40). The primary outcome was cesarean delivery.

**Results:**

Of 1236 included participants, 482 were not obese, while 754 had obesity: 288, 220, and 246 with class 1–3 obesity, respectively. Among the obese participants, the overall vaginal delivery rate was 69.9% (527/754). In a univariable analysis, participants with obesity were more likely to undergo cesarean delivery than their nonobese counterparts (*p* < 0.0001). In an adjusted multivariable analysis, participants with class 1–3 obesity were more likely to undergo cesarean delivery than their nonobese counterparts (class 1, adjusted odds ratio (aOR) 1.62, 95% confidence interval [CI] 1.12–2.36; class 2, aOR 2.30, 95% CI 1.55–3.41; class 3, aOR 2.96, 95% CI 2.02–4.33).

**Conclusions:**

People with obesity are more likely to undergo cesarean delivery during medically indicated late preterm induction, and risk increases with BMI class. However, the majority still deliver vaginally. Further studies are needed to understand differences in labor and induction physiology among pregnant people with obesity.

## 1 INTRODUCTION

The prevalence of obesity (class 1 obesity—body mass index [BMI] 30–34.9, class 2 obesity—BMI 35–39.9, and class 3 obesity—BMI ≥ 40) among reproductive‐aged people in the United States is 31%, and half of these people are class 2 or 3 obese [1]. Pregnant people with obesity are at increased risk for a number of pregnancy complications, including gestational diabetes mellitus (GDM), gestational hypertension (HTN), preeclampsia, large for gestational age newborns, stillbirth, and postpartum hemorrhage [1, 2]. People with obesity are also more likely to be recommended induction of labor (IOL) due to medical comorbidities [3, 4]. One study found that the rate of IOL increases with obesity class, with 30.4% for class 1, 32.5% for class 2, and 34% for class 3 and above compared to 28% among people without obesity [5].

People with obesity have been shown to have prolonged latent and active phases in labor compared to those without obesity, and are at increased risk of cesarean delivery, including during IOL [1, 6, 7, 8, 9, 10, 11]. Even when implementing standardized failed IOL criteria, studies have shown an increased risk of cesarean among people with obesity [5, 10]. Notably, prior studies have focused only on term pregnancies; data examining mode of delivery after preterm IOL, when delivery is almost always indicated and necessary, are sparse.

Whether people with obesity undergoing medically indicated late preterm IOL are at higher risk of cesarean than their counterparts without obesity is unknown. This study evaluates cesarean rates for patients undergoing medically indicated preterm IOL stratified by BMI category. We hypothesized that during late preterm medically indicated IOL, participants with obesity will have higher cesarean delivery rates than those without obesity.

## 2 MATERIALS AND METHODS

This is a secondary analysis of a multicenter, randomized trial of betamethasone versus placebo among pregnancies at risk for late preterm delivery, defined as delivery between 34 weeks and 0 days and 36 weeks and 6 days [12]. The parent trial, Antenatal Betamethasone for Women at Risk for Late Preterm Delivery (ALPS), was conducted at 17 university‐based clinical centers within the Maternal–Fetal Medicine Units Network of the *Eunice Kennedy Shriver* National Institute of Child Health and Human Development between 2010 and 2015. The parent trial included pregnant people with live, singleton, nonanomalous gestations at high risk for late preterm delivery due to labor or need for medically indicated delivery [12]. In the parent study, participants were included if an IOL was expected to start by 36 weeks 5 days, and among the exclusion criteria were unclear dating, having pregestational diabetes, or an expected delivery within 12 h.

For this secondary analysis, we included all participants in the parent trial who underwent a medically indicated preterm IOL. We excluded patients who underwent planned cesarean, had spontaneous labor, had a BMI <18.5 at their most recent measured weight prior to delivery, or had incomplete BMI data. We compared cesarean delivery rates among participants undergoing preterm IOL stratified by BMI class: no obesity (BMI 18.5–29.9), class 1 obesity (BMI 30–34.9), class 2 obesity (BMI 35–39.9), and class 3 obesity (BMI ≥ 40). BMI was calculated using the last measured weight prior to delivery. The primary outcome was cesarean birth. The indications for IOL and cesarean delivery were reviewed; one or multiple indications for both induction and cesarean were listed for each case.

Chi‐square was used for categorical data reported as frequencies and percentages; ANOVA or Wilcoxon was used for continuous variables reported as means with standard deviation (SD) or median with interquartile range (IQR), as appropriate. We fit multivariable regression models to determine the strength of association after adjusting for confounders identified in the literature and statistically significant baseline differences, including nulliparity, maternal age, chronic HTN (cHTN), GDM, and treatment group. A *p* value of less than 0.05 or a 95% confidence interval (CI) not crossing 1.0 was considered statistically significant. All statistical analyses were performed on SAS statistical software (Version 9.4).

The parent trial research protocol was approved by the Institutional Review Board of each clinical trial site. All patients in the parent trial provided voluntary written informed consent prior to study participation. Given the de‐identified data, this secondary analysis was deemed exempt and did not require separate oversight from our Institution Review Board.

## 3 RESULTS

**FIGURE 1:**
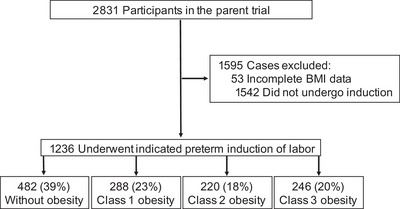
Study flowchart. BMI, body mass index.

Among the 2831 participants in the parent trial, 1236 participants underwent medically indicated late preterm IOL, with 482 (39%) without obesity, 288 (23%) with class 1 obesity, 220 (18%) with class 2 obesity, and 246 (20%) with class 3 obesity (Figure 1). Baseline demographics for the cohort are presented in Table 1. Multiple indications for induction were cited for each case, most commonly including preeclampsia and/or hypertension (688), premature rupture of membranes (254), fetal growth restriction (114), oligohydramnios (101), and nonreassuring fetal status (70). Other indications included cholestasis (40), other maternal disease (34), abruption (20), GDM (10), history of fetal demise/other serious pregnancy complication (7), chorioamnionitis (2), and other causes not described in the parent trial (44). The median gestational age at delivery for each group was 36 weeks (IQR 35, 37) and was statistically similar. Participants with class 1–3 obesity were more likely to be Black (20% with no obesity; 27%, 29%, and 37% with class 1–3 obesity, respectively), have cHTN (6% with no obesity; 13%, 21%, and 33% with class 1–3 obesity, respectively), or GDM (6% with no obesity; 13%, 18%, and 21% class 1–3 obesity, respectively) than their counterparts without obesity (all *p* < 0.0001). There were no differences in maternal age, insurance type, nulliparity, ethnicity, or tobacco use between groups. Neonates of participants with class 3 and class 4 obesity weighed more than the neonates of their normal weight counterparts (*p* < 0.0001). The incidence of low 5‐min APGAR scores (defined as 5‐min APGAR < 7) was not different between the groups (0.230).

**TABLE 1:** Baseline demographics among pregnant people with obesity undergoing late preterm induction of labor.

Demographic	Nonobese BMI (*N* = 482)	Class 1 obesity (*N* = 288)	Class 2 obesity (*N* = 220)	Class 3 obesity (*N* = 246)	*p* value
Pregnant person's age, mean ± SD	27.8 ± 6.1	28.4 ± 6.0	28.3 ± 5.6	28.9 ± 5.9	0.169
AMA, *n* (%)	72 (15.5%)	52 (18.4%)	36 (16.4%)	46 (18.9%)	0.606
Payer type, *n* (%)					0.094
Self‐pay/uninsured	25 (5.2)	12 (4.2)	6 (2.7)	14 (5.7)	
Private insurance	189 (39.2)	115 (39.9)	78 (35.4)	73 (29.7)	
Government‐assisted insurance	268 (55.6)	161 (55.9)	136 (61.8)	159 (64.6)	
Nulliparous, *n* (%)	209 (43.4)	114 (39.6)	76 (34.6)	108 (43.9)	0.112
Ethnicity, *n* (%)					0.266
Latinx	137 (28.5)	94 (32.4)	77 (35.1)	71 (29.1)	
Caucasian	344 (71.5)	194 (67.4)	142 (64.8)	173 (70.9)	
Race, *n* (%)					<0.0001
Black or African American	99 (20.5)	78 (27.1)	64 (29.1)	91 (37.0)	
White	295 (61.2)	168 (58.3)	128 (58.2)	127 (51.6)	
Asian	25 (5.2)	7 (2.4)	2 (0.9)	3 (1.2)	
Other/more than one	63 (13.1)	35 (12.2)	26 (11.8)	25 (10.2)	
Tobacco use, *n* (%)	68 (14.1)	35 (12.2)	26 (11.8)	35 (14.2)	0.752
cHTN, *n* (%)	27 (5.6)	37 (12.9)	47 (21.4)	81 (32.9)	<0.0001
GDM, *n* (%)	29 (6.0)	37 (12.9)	39 (17.7)	51 (20.7)	<0.0001
GA at delivery, median (IQR)	36 (35–37)	36 (35–37)	36 (35–37)	36 (35–37)	0.544
Neonatal weight at delivery in grams, mean ± SD	2450.2 ± 447.9	2527.6 ± 442.1	2608.6 + 460.8	2669.1 + 497.6	<0.0001
5‐min APGAR < 7, *n* (%)	9 (1.8)	13 (4.59)	7 (3.21)	7 (2.89)	0.230

Abbreviations: AMA, advanced maternal age; BMI, body mass index; cHTN, chronic hypertension; GA, gestational age; GDM, gestational diabetes mellitus; IQR, interquartile range.

**FIGURE 2:**
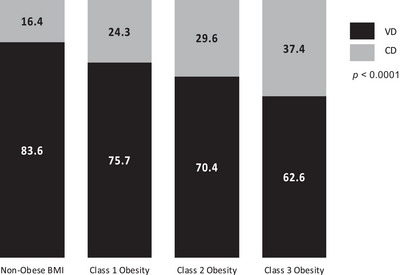
Univariate analysis of pregnant people's body mass index (BMI) and delivery method after preterm induction of labor. BMI, body mass index; CD, Cesarean delivery; VD, vaginal delivery.

Overall, 930 (75%) participants achieved a vaginal birth, while 306 (25%) participants underwent cesarean delivery. Among the obese participants, the overall vaginal delivery rate was 69.9% (527/754). In a univariable analysis, as BMI class increased, participants were more likely to undergo unplanned cesarean delivery, 79 (16.4%) with no obesity, 70 (24.3%) with class 1, 65 (29.6%) class 2, and 92 (37.4%) class 3 obesity (*p* < 0.0001). However, the majority of participants in each group had a vaginal birth, 83.6% among the no obesity group, 75.7 % among class 1 obesity, 70.4% among class 2 obesity, and 62.6% among class 3 obesity (Figure 2). Although multiple indications for cesarean were listed for each participant, the most common indications included nonreassuring fetal status (154, 50%) and labor dystocia (149, 49%). Other indications included preeclampsia and/or hypertension (59), malpresentation (15), chorioamnionitis (6), abruption (5), cord prolapse (4), other (2), prior cesarean (2), other maternal disease (1), and elective (1).

**FIGURE 3:**
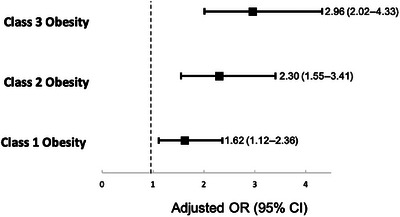
Multivariate logistic regression of pregnant people's body mass index (BMI) and delivery method after late preterm induction of labor adjusted for nulliparity, advanced maternal age (AMA), chronic hypertension (cHTN), gestational diabetes mellitus (GDM), and treatment group. Treatment group per the original trial was betamethasone administration versus placebo. CI, confidence interval; OR, odds ratio.

In a multivariable analysis adjusting for nulliparity, advanced maternal age, cHTN, GDM, and treatment group and using participants with no obesity as the reference, participants with class 1–3 obesity were more likely to undergo unplanned cesarean delivery (class 1, aOR 1.62, 95% CI 1.12–2.36; class 2, aOR 2.30, 95% CI 1.55–3.41; and class 3, aOR 2.96, 95% CI 2.02–4.33, Figure 3).

## 4 DISCUSSION

Our study found that pregnant people with obesity undergoing medically indicated late preterm IOL are at increased risk for unplanned cesarean with increasing BMI category compared to people without obesity, even after adjusting for confounders such as GDM, cHTN, and parity. Cesareans were largely performed for nonreassuring fetal status, highlighting fetal intolerance, and labor dystocia, highlighting parturition differences in people with obesity. However, the majority of participants (69.9%, 527/754) with obesity achieved a vaginal birth regardless of their BMI class, suggesting that when delivery is indicated, a vaginal trial of labor can be offered in the appropriate clinical context.

Our findings of increased cesareans among people with obesity undergoing preterm IOL are consistent with the existing literature for term IOL, where a similar association between BMI class and cesarean is appreciated [5, 11]. One large population‐based cohort study of singleton pregnancies found that rates of failed IOL and subsequent cesarean increased with BMI from 13% in people without obesity to 20.2%, 24.2%, and 29% in class 1–3 obesity, respectively [5]. A systematic review found increased odds of cesarean with increasing BMI; this review largely included studies that examined only patients at term as well as a few small studies that included both preterm and term births [11]. Our findings add to the literature by highlighting that this increased risk of failed IOL persists in the late preterm period, even though birth weights are smaller relative to term neonates.

Given that the majority of participants achieved a vaginal birth, IOL can be offered as the primary recommendation for patients with obesity who require late preterm delivery. Planned cesarean has not been shown to improve obstetrical outcomes in people with obesity, and the risk of cesarean may persist for pregnant patients with obesity after expectant management, as obesity is a risk factor for cesarean during spontaneous labor as well [13, 14, 15]. Though the majority of participants with obesity delivered vaginally, the increased risk of cesarean was notable. Cesarean delivery is associated with increased rates of hemorrhage, transfusion, venous thromboembolism, placental abnormalities in future pregnancies, and overall morbidity [9]. These risks associated with cesarean highlight the importance of optimizing preconception weight and weight gain during pregnancy.

Given the association between maternal obesity and labor dystocia noted in our cohort and at term, a number of studies have examined IOL techniques for people with obesity to identify whether there are benefits to any specific method. No benefits to any one agent, and no specific recommendations for IOL regimens in people with obesity, are currently available [16, 17, 18]. Given that 49% of the cesareans in our cohort occurred due to labor dystocia, and a breadth of literature notes prolonged labor curves for people with obesity [7, 8], further studies examining the optimal approach to IOL, and criteria for failure, in people with obesity are needed. Patients may also benefit from being counseled that obesity is a modifiable risk factor for risk of cesarean at the preconception visit.

Limitations for this study include that granular data on failed induction versus labor dystocia were not available, multiple indications were listed for cesareans, and we are unable to comment on some pertinent parameters related to induction, including the starting Bishop score, whether patients were undergoing trial of labor after cesarean, and the specific methods used for IOL. However, the fact that the parent trial allowed pragmatic management at the discretion of the provider makes the findings more generalizable. Other strengths included that, as an analysis from a prospective randomized controlled trial, the data are robust. The large cohort of participants received care at geographically diverse academic medical centers, also increasing the generalizability of our findings. Finally, we used data on maternal BMI at the time of delivery instead of prepregnancy BMI, as this is more pertinent to counseling at the time of delivery.

## 5 CONCLUSIONS

While obesity and obesity class were associated with higher rates of cesarean compared with pregnant individuals without obesity, the majority (69.9%, 527/754) of pregnant people with obesity undergoing late preterm IOL delivered vaginally, and thus obesity should not necessarily deter providers from offering IOL when there are no obstetric contraindications. Given increasing rates of obesity among people of reproductive age, further studies are needed to understand differences in labor and induction physiology among pregnant people with obesity.

## CONFLICT OF INTEREST STATEMENT

The authors declare no conflicts of interest.

## ETHICS STATEMENT

The parent trial research protocol was approved by the Institutional Review Board of each clinical site where the randomized control study took place. This secondary analysis was exempt from review given the deidentified nature of the data used for secondary analysis.

## Data Availability

The authors confirm that data supporting the findings of this study are available within the article.
